# Single-cell transcriptional analysis reveals allergen-specific signatures in human **γδ** T cells

**DOI:** 10.1172/jci.insight.191359

**Published:** 2025-04-17

**Authors:** Kendall Kearns, Sloan A. Lewis, Esther Dawen Yu, Adam Abawi, Eric Wang, Synaida Maiche, Monalisa Mondal, Pandurangan Vijayanand, Grégory Seumois, Bjoern Peters, Alessandro Sette, Ricardo Da Silva Antunes

**Affiliations:** 1 Center for Infectious Disease and Vaccine Research, La Jolla Institute for Immunology (LJI), La Jolla, California, USA.; 2 Biomedical Sciences Graduate Program and; 3 Department of Medicine, Division of Infectious Diseases and Global Public Health, UCSD, La Jolla, California, USA.

**Keywords:** Cell biology, Immunology, Adaptive immunity, Allergy, T cells

## Abstract

The role of gamma-delta T (γδ T) cells in immune responses to common allergens is poorly understood. Here, we utilized single-cell (sc) transcriptomic analysis of allergen-reactive γδ T cells in humans to characterize the transcriptional landscapes and TCR repertoires in response to cockroach (CR) and mouse (MO) allergens. Using a potentially novel activation-induced marker (AIM) assay that allows detection of γδ T cells combined with scRNA sequencing and TCR repertoire analysis, we identified both shared and allergen-specific γδ T cell activation patterns and gene expression profiles. While CR extract activated both Vδ1 and Vδ2 subsets, MO extract primarily stimulated Vδ2 cells. Our analysis revealed allergen-specific clusters with distinct functional signatures, including enhanced inflammatory responses and cytotoxic effector functions in MO-specific γδ T cells and natural killer cell–mediated immunity and IFN-γ signaling in CR-specific populations. Comparison of allergic and nonallergic individuals highlighted differences in gene expression and TCR repertoires, including a higher *IFNG* expression in the CR-allergic compared with nonallergic cohorts, suggesting that phenotypic and functional differences are associated with γδ T allergen responses. This study provides insights into the cellular and molecular heterogeneity and functionality of allergen-reactive γδ T cells, offering a foundation for understanding their role in allergic diseases and potential therapeutic interventions.

## Abstract 2

Study reveals how specialized cells bridinging the adaptive and innate immune systems respond differently to common allergens, providing new insights into allergic disease mechanisms,

## Graphical abstract

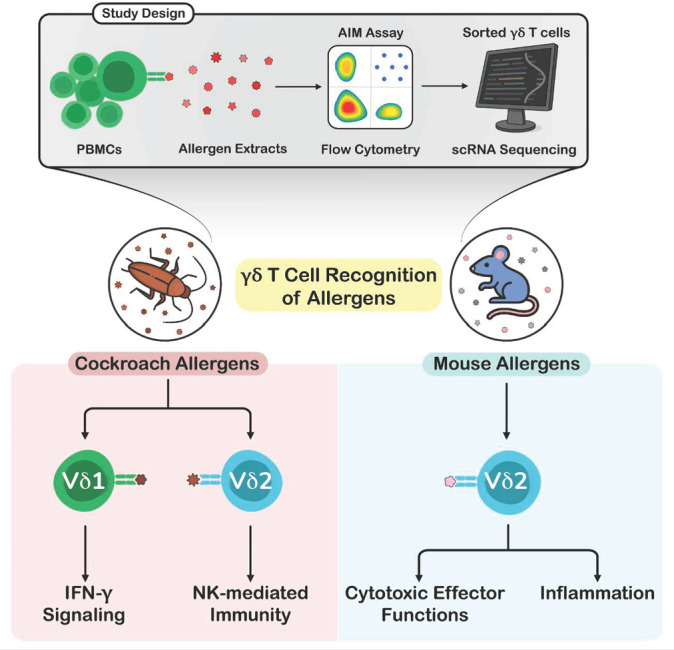

## Introduction

Gamma-delta T (γδ T) cells, uniquely positioned at the interface of innate and adaptive immunity, have emerged as a population of particular immunological interest, being involved in the initiation and regulation of many immunopathologies, including cancer, autoimmune diseases, and allergy (1–5). While CD4^+^ T helper 2 (Th2) cells and IgE-producing B cells (6, 7) have well-established roles in the pathogenesis of allergic diseases, the contribution of γδ T cells is still being unraveled.

γδ T cells are a distinct subset of T lymphocytes characterized by a TCR composed of γ and δ chains, in contrast with the αβ TCR found on conventional T cells (8). γδ T cells are known for their rapid response to stress signals and their ability to recognize nonpeptide antigens without the need for conventional antigen presentation (9, 10). While present in peripheral blood, γδ T cells are particularly abundant in epithelial tissues, including the skin, respiratory tract, and gastrointestinal mucosa — sites that are crucial in allergic responses (11, 12). This strategic localization, combined with their capacity for rapid inflammatory cytokine production and cytotoxic activity, positions γδ T cells as potential key players in the initiation, progression, and regulation of allergic responses (2, 13).

Published reports on γδ T cells in allergy suggest both pro-inflammatory roles (e.g., producing IL-17 in asthma) and regulatory functions (e.g., suppressing airway inflammation and promoting oral tolerance) (14–17). These seemingly contradictory findings regarding the role of γδ T cells in allergic responses may be explained by the existence of different γδ T cell subsets associated with different allergen responsiveness, functional and transcriptional programs, and TCR gene expression (18). In humans, γδ T cells are broadly categorized into 2 main subsets based on their δ chain usage: Vδ1 and Vδ2 cells (19). Vδ1 cells are predominantly found in mucosal tissues (20, 21), while Vδ2 cells, which typically pair with the Vγ9 chain, are the major circulating subset and are known for their rapid production of pro-inflammatory cytokines in response to phosphoantigens (22, 23). However, a comprehensive understanding of how different γδ T cell subsets respond to specific allergens, their immune profile, and how these responses differ between allergic and nonallergic individuals remains elusive.

The reactivity of γδ T cells to common allergens and their subsequent functional profiles remain largely unknown. We recently reported the development of ex vivo assays using peripheral blood to detect allergen-reactive human γδ T cells (24). Our findings revealed that human γδ T cells become activated in response to common environmental allergens, producing allergen-specific responses and exhibiting a Th1-polarized profile in allergic donors. In this study, to further explore the cellular and molecular heterogeneity and functional diversity of allergen-reactive γδ T cells, we defined the immune signatures of allergen-reactive γδ T cells responding to common allergens (i.e., cockroach, CR; and mouse, MO), using single-cell RNA sequencing (scRNA-Seq) to provide a high-resolution map of the transcriptional landscapes of γδ T cells.

## Results

### γδ T cell subset frequencies vary by allergen stimulation.

**Figure 1:**
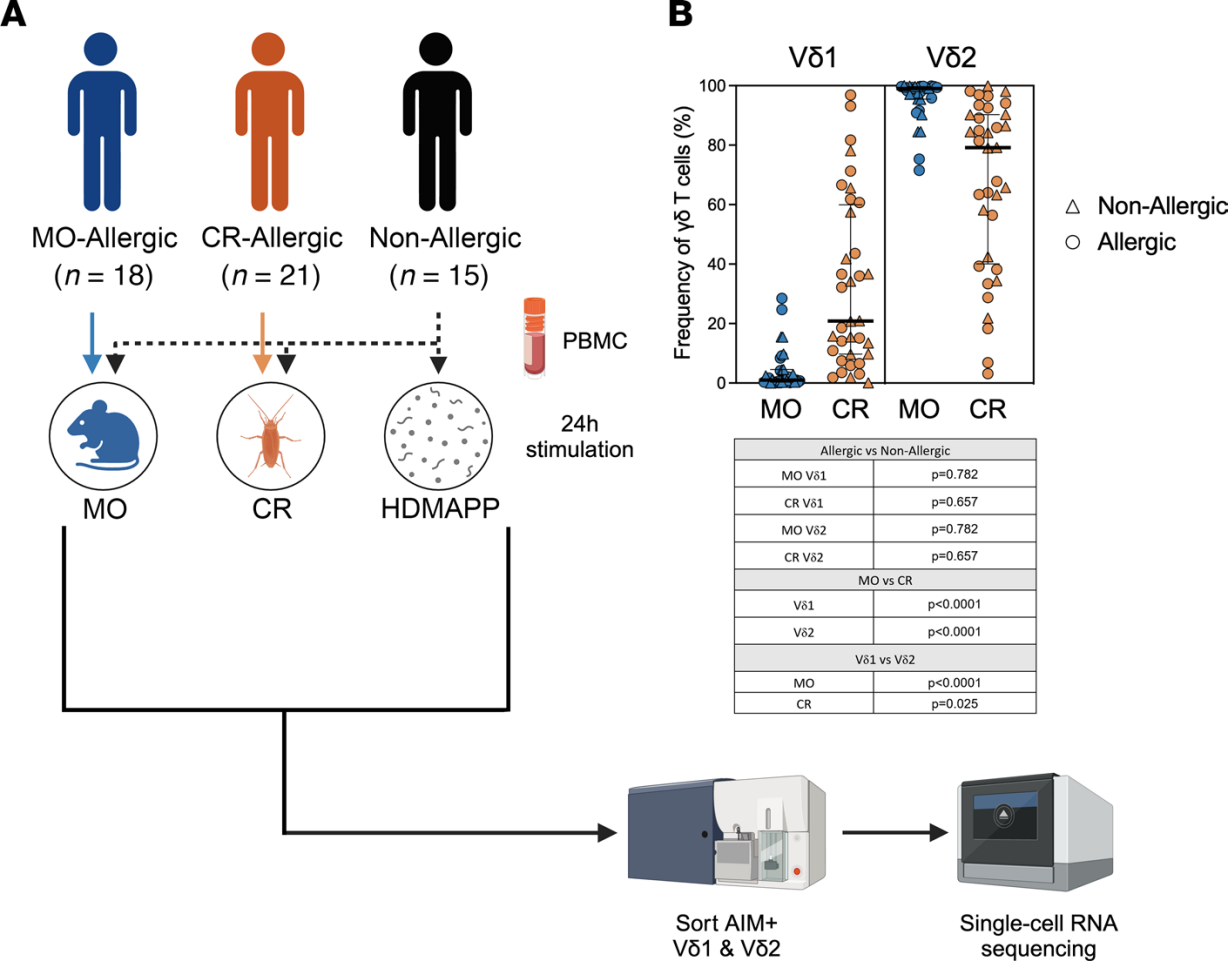
Experimental strategy. (**A**) PBMCs from cockroach-allergic, mouse-allergic, and nonallergic individuals were stimulated for 24 hours with cockroach extract, mouse extract, or either cockroach extract, mouse extract, or HDMAPP, respectively. AIM^+^ (CD69^+^CD137^+^) Vδ1 and Vδ2 cells were isolated and sequenced. Figure created with BioRender.com. (**B**) Frequency of Vδ1 and Vδ2 subsets of AIM^+^ γδ T cells within each stimulated sample. Table insert depicts the *P* values for each of the different statistical comparisons, performed using the Mann-Whitney test (allergic vs. nonallergic proportions) or the Wilcoxon test (Vδ1 vs. Vδ2 proportions). *P* < 0.05 was considered statistically significant.

We previously developed an activation-induced marker (AIM) assay to detect human γδ T cell responses directly ex vivo, measuring upregulation of cell surface markers CD69 and CD137 (24). This method effectively identified γδ T cell reactivity to HDMAPP, a potent γδ T cell activator, and successfully detected TCR-dependent allergen-reactive γδ T cells. To determine how stimulation with different allergens affects the transcriptional profiles of γδ T cells, and to identify potential differences between nonallergic and allergic donors in allergen-reactive γδ T cell gene expression and TCR repertoires, using a cohort of 53 donors, we stimulated peripheral blood mononuclear cell (PBMC) samples from CR-allergic, MO-allergic, and nonallergic donors with CR extract, MO extract, and/or HDMAPP (Figure 1A). Donors allergic to the different allergens were defined by allergen-specific IgE titers of more than 0.35 kUA/L. Detailed clinical and demographic data are described in Supplemental Table 1; supplemental material available online with this article; https://doi.org/10.1172/jci.insight.191359DS1 CR and MO extracts were generated as previously described (24). These allergens were chosen because of their clinical relevance and the distinct nature of the antigens they contain, potentially activating different subsets of γδ T cells (24).

AIM^+^ (CD69^+^CD137^+^) Vδ1 and Vδ2 cells were captured by fluorescence-activated cell sorting (FACS; Supplemental Figure 1) followed by scRNA-Seq (see Methods). FACS analysis of cell surface expression revealed that most of the activated γδ T cells from the PBMC samples were Vδ2 cells, as expected since Vδ2 cells are the most common γδ T cell subset in the blood (14, 22) (Figure 1B). However, significant numbers of Vδ1 cells were also observed, but almost exclusively in CR-stimulated samples, suggesting that CR extract, unlike MO extract, is recognized by both Vδ1 and Vδ2 cells. This pattern was observed across all groups without significant differences in the frequency of γδ T cells between allergic and nonallergic donors (Figure 1B, table insert), which corroborated our previous findings (24).

### Stimulation with allergen drives clustering of scRNA-Seq data.

**Figure 2:**
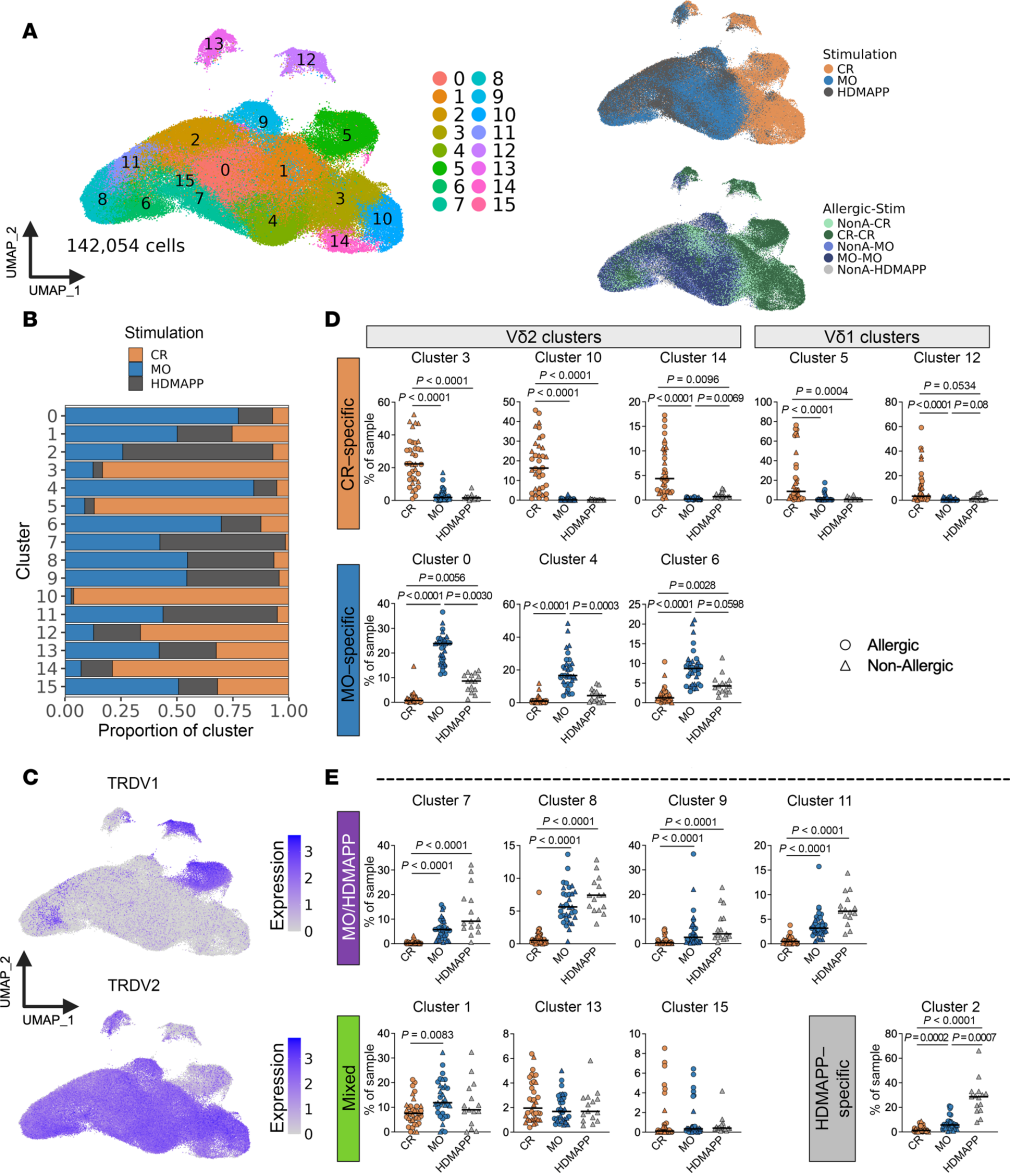
Stimulation with allergen drives clustering of scRNA-sequencing data. (**A**) Uniform manifold approximation and projection (UMAP) visualizations of scRNA-sequencing data colored by cluster (left), stimulation (top right), or allergic-stimulation condition (bottom right). (**B**) Proportion of each cluster attributable to CR-, MO-, or HDMAPP-stimulated samples. (**C**) UMAP visualizations of TRDV1 (top) or TRDV2 (bottom) expression. (**D** and **E**) Graphs show the percentage of cells from each stimulus found in each cluster and the attribution of antigen specificity to each individual cluster. Kruskal-Wallis test with Dunn’s multiple comparisons test was performed to determine if significantly more cells from 1 stimulus were present in each cluster compared with the other stimuli, thus allowing us to identify stimulus-specific clusters. *P* < 0.05 was considered statistically significant.

Following scRNA-Seq, we filtered for cells that passed quality control metrics (see Methods), resulting in a total of 142,052 cells. Unbiased clustering based on gene expression revealed 16 distinct clusters of allergen-reactive γδ T cells (Figure 2A and Supplemental Figure 2A). Using both manual annotation and functional enrichment analysis, we identified distinct biological signatures and several clusters of interest, including clusters with a pro-eminent IFN-γ signaling signature (cluster 10), NK cell–like features (cluster 5), and cytotoxic function (cluster 4) (Figure 2, Supplemental Figure 2A, and Supplemental Table 2). Separating the cells by antigenic stimulus showed clear divergence within UMAP space (Figure 2A). Several clusters were mostly composed of CR-reactive cells, while other clusters consisted of a more balanced mix of MO- and HDMAPP-reactive cells (Figure 2B). The clusters were also clearly differentiated by their expression of *TRDV1* (clusters 5, 12) or *TRDV2* (remaining clusters) (Figure 2C and Supplemental Figure 2B), validating the surface expression FACS data, in which Vδ1 cells were found in the CR-stimulated samples (approximately 20% of total CR-specific γδ T cells) while essentially absent in the MO-stimulated samples (Figure 1B).

To define the antigen specificity of each individual cluster, we analyzed the distribution of stimulus-specific responses across all stimulated samples (Figure 2D). We further divided these clusters based on TCRδ (TRDV) gene expression patterns (Supplemental Figure 2, A and B). This approach revealed significantly enriched and distinct stimulus-specific clusters: 3 Vδ2 CR-specific clusters (clusters 3, 10, and 14), 2 Vδ1 CR-specific clusters (clusters 5 and 12), and 3 Vδ2 MO-specific clusters (clusters 0, 4, and 6) (Figure 2D). Additionally, we identified clusters responding to multiple stimuli (such as MO/HDMAPP and mixed clusters) as well as 1 cluster that responded exclusively to HDMAPP stimulation (Figure 2E).

After analyzing the top genes expressed in each cluster (Supplemental Figure 2C) and their functional pathways (Supplemental Figure 2D), we observed several shared patterns within clusters responding to the same stimulant. While HDMAPP-stimulated clusters were often associated with a stronger activation signature compared with other clusters, we focused our downstream analyses on allergen responses to identify potential differences between allergic and nonallergic donors in CR- and MO-specific clusters only.

### MO- and CR-specific clusters are dominated by Vγ9Vδ2 gene usage with more diversity in CR samples.

We next examined the TCR repertoire of our stimulus-specific clusters. As we did not use custom γδ TCR primers for the scRNA-Seq experiment, we utilized TRUST4 (25) to reconstruct the TCR repertoire from gene expression data. We examined the TCR repertoire using paired V(D)JC gene combinations to define TCR clonotypes (26) instead of the typical complementarity-determining region 3 amino acid sequence. To account for variations in the number of cells with paired TCR genes across samples, we downsampled each sample to the median TCR count per cohort, reducing potential bias (Methods and Supplemental Table 3). This standardization preserved the original diversity patterns for each cohort (Supplemental Figure 3A).

Regarding MO-specific clusters, we found that cluster 6 had slightly more pronounced clonal expansion than the other clusters (Supplemental Figure 3B). Twelve unique clones shared across all 3 MO-specific clusters (Supplemental Figure 3B) comprised over 70% of the repertoire in each of the 3 clusters, most of which were associated with the *TRGV9/TRDV2* gene combination (Table 1). In terms of clonal expansion of the TCR repertoire across the CR-specific clusters, only the Vδ2 clusters had “large” expanded clones (Supplemental Figure 3C), suggesting a more restricted gene pairing within the Vδ2 repertoire, as expected, since the majority of Vδ2 cells also express Vγ9 (14, 22). Additionally, there were largely separate unique clones shared within the Vδ1 and Vδ2 clusters (Supplemental Figure 3C and Table 2). Five unique clones, all Vγ9Vδ2, were shared across the 3 Vδ2 clusters and comprised over 50% of the repertoire for each cluster. Eight clones were shared within the Vδ1 clusters but comprised only approximately 34% of the cluster 5 repertoire compared with 78% of the cluster 12 repertoire. Thus, there is more diversity within both Vδ1 and Vδ2 TCR repertoires responsive to CR extract compared with the MO extract.

We next examined the TCR repertoire for allergic and nonallergic samples separately. For the MO-specific clusters, the 12 shared most abundant clones (Table 1) were largely shared between allergic and nonallergic samples (Supplemental Figure 3D), but a greater proportion of large expanded clones was associated with allergic samples compared with nonallergic cohorts (Supplemental Figure 3F). CR-specific clusters, particularly clusters 5, 3, and 10, were not associated with shared clones to the same extent as that of the MO-specific clusters (Supplemental Figure 3E). We also observed only minor differences between cohorts for clonal expansion (Supplemental Figure 3G).

Finally, we examined the specific pairings of variable TCRγ (TRGV) and TRDV genes. As expected, based on the UMAP clustering (Figure 2), no gene pairs containing TRDV1 were observed in the MO-specific clusters, and the majority of pairings were *TRGV9*/*TRDV2* in both allergic and nonallergic samples (Supplemental Figure 3H). For the CR-specific clusters, the specific gene pairs utilized by the allergic and nonallergic cohorts were similar, with some bias for TRGV3*01/TRDV1*01 in allergic samples and TRGV2*01/TRDV1*01 in nonallergic samples (Supplemental Figure 3I). Overall, these findings indicate that the repertoires are dominated by Vγ9Vδ2 with greater diversity in the CR-specific clusters and potential differences in expansion across the cohorts.

### MO-specific clusters are defined by distinct cytokine production and activation signatures.

**Figure 3:**
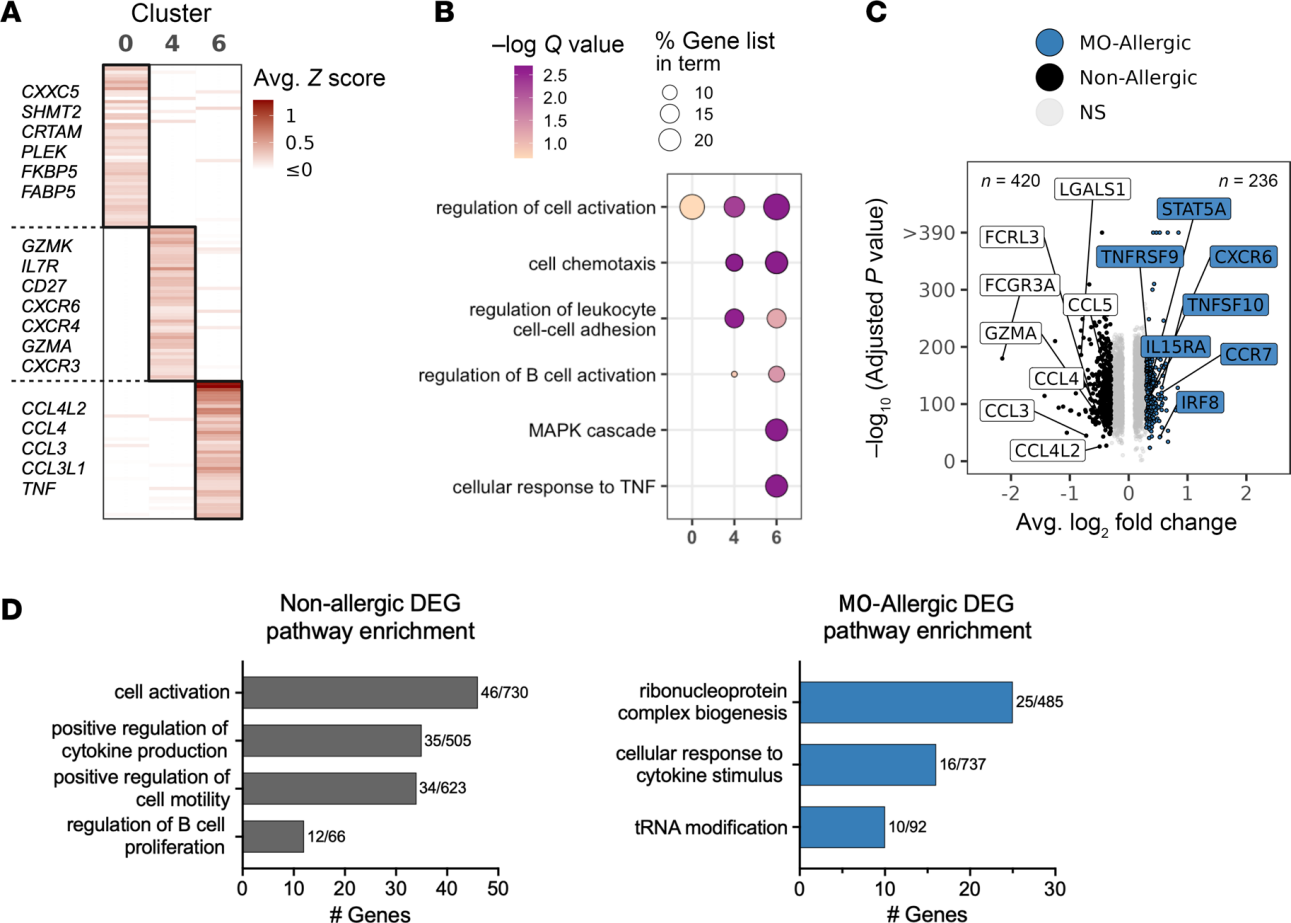
MO-specific clusters are defined by distinct cytokine production, activation, and dominant Vγ9Vδ2 TCR pairing. (**A**) Heatmap of average scaled expression of the top 50 genes per cluster. Genes listed at left are unique to that specific cluster. (**B**) Pathway enrichment of genes with a log_2_ fold-change > 0.5 in each cluster compared with all other clusters (i.e., not solely MO-specific clusters). (**C**) Volcano plot showing DEGs between allergic and nonallergic samples stimulated with MO extract within MO-specific clusters. Selected genes annotated. (**D**) Pathway enrichment of DEGs found upregulated in nonallergic (left) and allergic (right) samples. The number of DEGs out of the total number of genes within each pathway are noted.

Analysis of the 3 MO-specific clusters (clusters 0, 4, and 6) showed distinct gene expression profiles (Figure 3A), as further verified by Metascape pathway enrichment analysis (27) (Figure 3B). While all MO-specific clusters shared pathways related to cell activation, likely reflective of the allergen stimulation, clusters 4 and 6 specifically shared pathways involved in leukocyte cell-cell adhesion. Notably, cluster 6 displayed a unique chemokine and cytokine signature with high *TNF* expression (Figure 3, A and B), consistent with our previous observations of elevated TNF-α production in MO-stimulated samples (24).

Initial analysis of allergic and nonallergic samples stimulated with the MO extract revealed no significant differences in the proportion of cells found in each of the 3 MO-specific clusters (Supplemental Figure 4A). This suggests that the unbiased cell clusters do not capture differences between disease states. However, further differentially expressed gene (DEG) analysis (Figure 3C and Supplemental Table 4) revealed distinct patterns between MO-allergic and nonallergic samples. Specifically, we noted upregulation of genes such as *IL15RA*, *CCR7*, and *CXCR6* in the allergic cohort and *GZMA*, *FCGR3A*, and various chemokines and cytokines in the nonallergic cohort (Figure 3C). We calculated module scores using DEGs to evaluate their average expression patterns across clusters. DEGs linked to allergic samples showed consistent expression levels throughout the 3 MO-specific clusters. In contrast, DEGs associated with nonallergic samples exhibited slightly higher expression in clusters 4 and 6 (Supplemental Figure 4B).

Of the Gene Ontology (GO) Biological Processes pathways, we found a significant difference (*P* = 0.0046) in the top enriched pathways between the allergic and nonallergic groups (Supplemental Figure 4C). Upregulated genes within the nonallergic samples were strongly associated with cell activation and cytokine production, while allergic samples showed upregulation of genes associated with ribonucleoprotein complex biogenesis and cellular response to cytokine stimulus (Figure 3D). In summary, although there were no differences in frequency, we observed qualitative differences in the gene signatures between MO-allergic and nonallergic samples.

### CR-specific Vδ1 and Vδ2 clusters are distinct in gene expression with enhanced IFNG^+^ Vδ2 signature in allergic samples.

**Figure 4:**
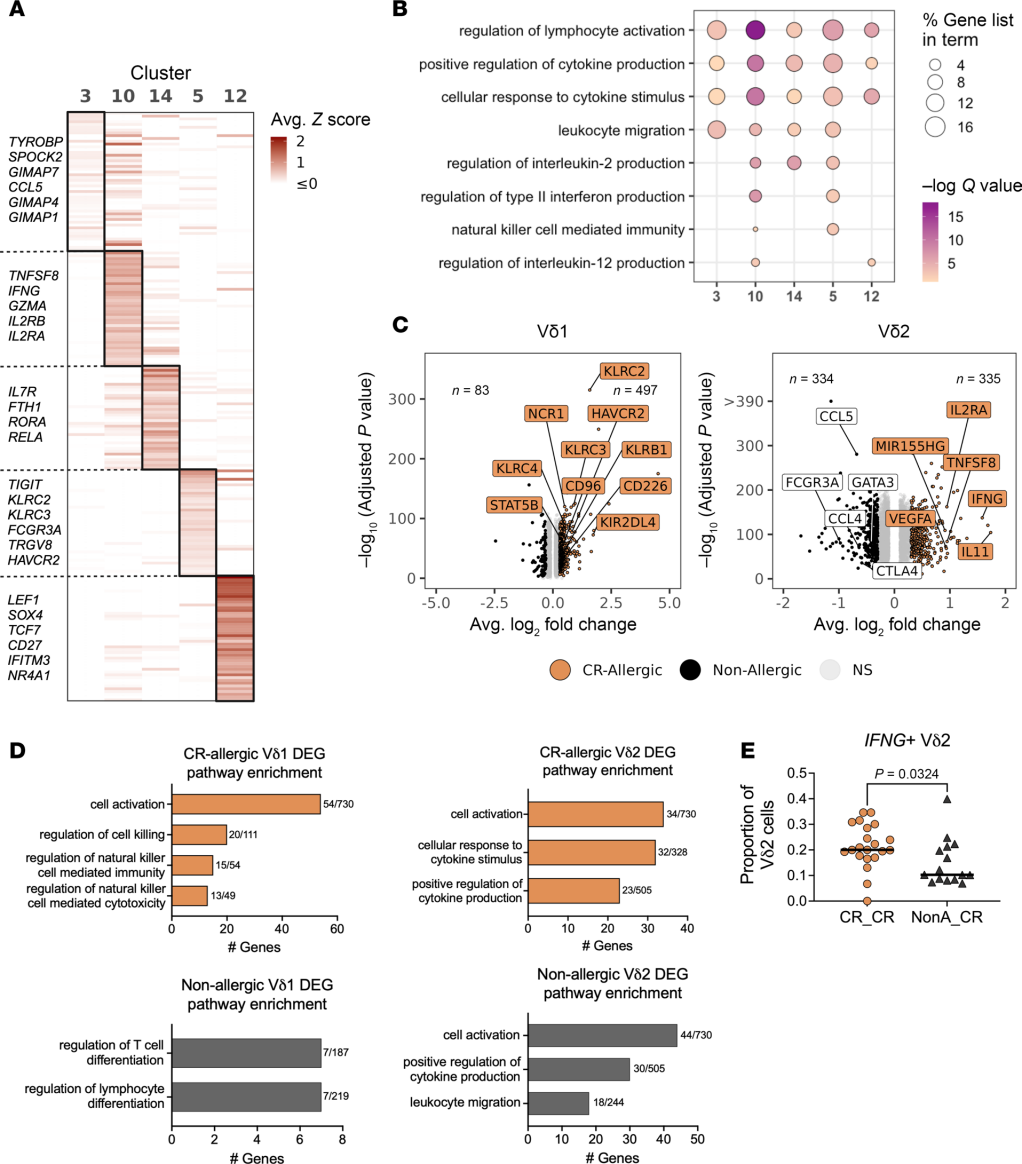
CR-specific Vδ1 and Vδ2 clusters are distinct in gene expression and TCR gene usage. (**A**) Heatmap of average scaled expression of the top 50 genes per cluster. Genes listed at left are unique to that specific cluster. (**B**) Pathway enrichment of genes with a log_2_ fold-change > 0.5 in each cluster compared with all other clusters (i.e., not solely CR-specific clusters). (**C**) Volcano plots showing differentially expressed genes between allergic and nonallergic samples stimulated with CR extract for Vδ1 (left) and Vδ2 (right) cells within CR-specific clusters separately. Selected genes annotated. (**D**) Pathway enrichment of DEGs found upregulated in allergic (top) and nonallergic (bottom) samples for CR-specific Vδ1 (left) and Vδ2 (right) clusters. The number of DEGs out of the total number of genes within each pathway are noted. (**E**) Proportion of Vδ2 cells with IFNG expression (>1) separated by allergic status (right).

We analyzed the CR-specific clusters using the same approach as for the MO-specific clusters. The gene expression profiles of the 5 clusters were not as distinct as the MO-specific clusters, with some genes expressed in multiple clusters (Figure 4A). The pathway enrichment analysis revealed that all 5 CR-specific γδ T cell clusters were significantly enriched for genes involved in the regulation of lymphocyte activation and cytokine production (Figure 4B). However, distinct pathways were selectively enriched in specific clusters, such as NK cell–mediated immunity and regulation of IL-2, type II IFN, and IL-12 production in clusters 5 and 10.

When we examined the proportion of allergic and nonallergic samples in each of the 5 CR-specific clusters, we found no significant differences (Supplemental Figure 5A). To identify potential qualitative differences between allergic and nonallergic samples, we performed DEG analysis as described above (Figure 4C and Supplemental Table 5). Since Vδ1 and Vδ2 cells are known to comprise transcriptionally distinct subsets (28–30), analyses for CR-specific Vδ1 and Vδ2 clusters were conducted separately. Remarkably, Vδ1 cells from allergic individuals exhibited a significantly higher number of upregulated genes (*n* = 497) compared with nonallergic individuals (*n* = 83). Based on module scores, we identified higher scores in cluster 5, while those using DEGs associated with nonallergic samples revealed higher scores in cluster 12 (Supplemental Figure 5B). Importantly, we also found significantly different pathway enrichment between the allergic and nonallergic cohorts for Vδ1 cells (*P* < 0.0001) (Supplemental Figure 5C), which were mostly associated with cell activation and processes related to NK cell–mediated immunity in allergic participants (Figure 4D), while nonallergic-associated DEGs were enriched for T cell differentiation pathways (Figure 4D).

In contrast, for the Vδ2 clusters, the numbers of DEGs were comparable between allergic and nonallergic samples (Figure 4C). Allergic DEGs were similarly expressed across clusters based on module scores, while cluster 3 had higher module scores compared with the other clusters in nonallergic samples (Supplemental Figure 5B). The top enriched pathways in Vδ2 cells were not significantly different between cohorts (Supplemental Figure 5C), with a strong signature of genes associated with cell activation and cytokine-related pathways (Figure 4D). Of relevance, along with *IL11* and other cytokines, *IFNG* was one of the top genes upregulated in allergic samples, supporting observations from our previous work (24). Further examination of the proportion of Vδ2 cells with *IFNG* expression in the allergic and nonallergic cohorts revealed a significant enrichment of *IFNG*^+^ Vδ2 cells in the allergic samples (Figure 4E), suggesting recall T cell responses with early production of IFN-γ (31, 32).

Overall, these findings corroborate our earlier research, which demonstrated that both Vδ1 and Vδ2 cells are activated by CR extract with a particular enhanced IFN-γ signature in allergic donors.

## Discussion

Here, we applied a potentially new γδ T cell–specific AIM assay (24) to detect and isolate antigen-specific γδ T cells directly ex vivo, thus allowing their detailed characterization by scRNA-Seq and TCR profiling. We uncovered different γδ T functional subsets defined by 4 main distinguishing features: first, their responsiveness to different allergenic stimuli; second, their expression of distinct transcriptional profiles as a function of subsets; third, the expression of distinct transcriptomic signatures as a function of allergic status; and fourth, their differential expression of specific TCR genes. We believe this is the first detailed demonstration using transcriptomic resolution of diverse γδ T functional subsets in response to different stimuli, revealing their potential functional heterogeneity across different biological contexts.

We found that distinct subsets of γδ T cells were preferentially activated by different allergens, with CR extract activating both Vδ1 and Vδ2 cells and MO extract primarily activating Vδ2 cells. This is consistent with previous reports indicating that γδ T cell subsets can respond to different antigens (15, 19). The preferential activation of Vδ1 cells by CR extract is particularly intriguing, as these cells have been implicated in mucosal immunity and may play a crucial role in the response to inhaled allergens (12, 33).

In terms of expression of distinct transcriptomic profiles associated with the different subsets, the clustering analysis of scRNA-Seq data revealed both shared and distinct transcriptional profiles of Vδ1 and Vδ2 cells associated with different allergen stimulations. Notably, MO-specific clusters exhibited strong activation signatures and elevated expression of chemokines and cytokines, particularly *TNF*. This finding corroborates our previous work (24) and suggests that the MO allergen may induce a more pronounced inflammatory response as compared with the CR allergen. This could be at least in part related to different antigenic compounds (34, 35) being contained in the CR and MO extracts. The CR-specific clusters were enriched for pathways related to lymphocyte activation and cytokine production, as well as upregulation of genes involved in NK cell–mediated immunity and regulation of IL-2, type II IFN, and IL-12 production. Thus, CR-specific Vδ1 and Vδ2 responses are more complex than their MO counterparts. This is consistent with the higher complexity of the CR extracts compared with the MO urine extract utilized in this study (24, 36, 37).

In terms of expression of distinct signatures associated with allergic versus nonallergic status, our study is the first to our knowledge to report on transcriptional profiles and TCR repertoires of allergen-reactive γδ T cells in allergic and nonallergic individuals. While the frequencies of allergen-specific γδ T cells were overall similar in the allergic and nonallergic cohorts, we uncovered key qualitative differences in their gene expression profiles and TCR repertoires. In MO-stimulated samples, allergic individuals showed a central memory phenotype with high expression of *CCR7* and *CXCR6*, suggesting that they may be primed to rapidly migrate into tissues (38, 39). In nonallergic individuals, MO responses showed upregulation of genes associated with cell activation and regulation of cytokine production and B cell proliferation.

For CR-stimulated samples, we observed distinct patterns in Vδ1 and Vδ2 cells. Vδ1 cells from allergic individuals showed upregulation of genes related to cell activation and NK cell–like functions, indicating a potentially heightened innate-like response in allergic individuals (40). In Vδ2 cells, we found a significant enrichment of *IL11*- and *IFNG*-expressing cells in allergic samples, supporting our previous findings (24). A more activated, pro-inflammatory phenotype of γδ T cells in allergic individuals may contribute to the pathogenesis of CR allergy. Alternatively, the enrichment of *IFNG*-expressing Vδ2 cells in allergic individuals might play a protective role by counterbalancing the classical Th2-polarized responses associated with allergic settings (41, 42). Further investigation of the potential role of these cells in the modulation of allergic disease or their potential as therapeutic targets is warranted.

Finally, the analysis of TCR repertoires revealed patterns of clonal expansion and gene usage. Specifically, in MO-specific clusters, we observed a predominance of *TRGV9*/*TRDV2* gene combinations, consistent with the known prevalence of this pairing in peripheral blood Vδ2 cells (30, 43). In contrast, the CR-specific response showed a more diverse repertoire and gene usage, with distinct patterns in Vδ1 and Vδ2 populations. Vδ2 cells in CR-specific clusters showed evidence of larger clonal expansions compared with Vδ1 cells. This could indicate that while CR extract activates both subsets, Vδ2 cells may undergo more robust proliferation in response to the CR stimulus. The slightly increased clonal expansion observed in CR allergic samples may indicate the presence of allergen-specific γδ T cell populations that have undergone in vivo clonal selection.

Our study has several limitations. First, the specific antigens within the allergen extracts that trigger recognition remain undefined. γδ T cells possess unique antigen recognition mechanisms compared with conventional αβ T cells, which may mitigate some abundance-related biases associated with AIM assays (44). The evidence of high clonal expansion in vivo suggests our assay can capture responses to less abundant antigens if they stimulate a high-affinity T cell response. However, some less prevalent but potentially functionally significant antigen-specific responses may still be underrepresented. Identifying the specific components of CR and MO extracts responsible for these effects could provide insights into γδ T cell antigen recognition and lead to novel therapeutic approaches. Second, the number and timing of the samples collected are limited because of the exploratory nature of the study and limited sample availability. In future studies, it will be of interest to include a larger number of individuals, with longitudinal sampling and defined associated clinical outcomes. Future studies would also benefit from validation of gene signatures through functional assays. Furthermore, whether the observed transcriptional differences are reflective of different tissue-homing properties, and whether the same patterns also apply to tissue-resident cells, have not been evaluated. Another limitation of this study is the use of TRUST4 to reconstruct the γδ TCR repertoire from gene expression data, which captures only a fraction of the diversity obtainable through targeted sequencing. Finally, the functional relationship of the different subsets defined herein with other components of the allergic immune response, such as IgE-producing B cells or Th2 cells, has also not been addressed in the present study.

In conclusion, our study provides a comprehensive analysis of allergen-reactive γδ T cell subsets at the single-cell level, revealing allergen-specific and allergy status–dependent differences in gene expression and TCR repertoires. These findings contribute to our understanding of the immunological basis of allergic responses and may inform the development of more targeted and effective treatments for allergies.

## Methods

### Sex as a biological variable.

Our study examined male and female participants, and similar findings are reported for both sexes.

### Experimental model and participant details.

The study cohort recruited for this study included 53 donors (Supplemental Table 1). A total of 37 donors were sensitized to either MO (*n* = 17) or CR (*n* = 20) while 1 donor was sensitized to both MO and CR, defined by allergen-specific IgE titers of more than 0.35 kUA/L. A total of 15 nonallergic healthy controls exhibited undetectable IgE titers (<0.01 kUA/L) for both MO and CR allergens. Demographic data associated with each donor are described in Supplemental Table 1. All donors were from San Diego, California, USA. Adults of all races, ethnicities, ages, and sexes were eligible to participate. Each participant was assigned a study identification number with clinical information recorded. Clinical symptoms of allergy were collected by questionnaire-based survey, and IgE titers were determined from plasma using Phadia’s ImmunoCAP assay (Thermo Fisher Scientific).

### Generation of MO and CR allergen extracts.

MO and CR extracts were generated as previously described (24). Briefly, mouse urine (mixed sex pooled, unfiltered) was purchased from CliniSciences, lyophilized, and subsequently resuspended in PBS at 5.7 mg/mL (confirmed by Pierce BCA Protein Assay Kit, Thermo Fisher Scientific). CR extract was obtained from German cockroach frass (cockroach debris containing body parts, fecal matter, and egg cases) and manufactured in-house at the La Jolla Institute for Immunology using established protocols described elsewhere (45).

### PBMC isolation and thawing.

PBMCs were isolated from whole blood by density gradient centrifugation according to manufacturer instructions (Ficoll-Hypaque, Amersham Biosciences) and cryopreserved for further analysis. Cryopreserved PBMCs were quickly thawed by incubating each cryovial at 37°C for 2 minutes, and cells were transferred into 9 mL of cold medium (RPMI 1640 with l-glutamine and 25 mM HEPES from Omega Scientific), supplemented with 5% human AB serum (GemCell), 1% Penicillin Streptomycin (Gibco), 1% Glutamax (Gibco), and 20 U/mL Benzonase Nuclease (MilliporeSigma). Cells were centrifuged at 300*g* and resuspended in medium to determine cell concentration and viability using trypan blue and a Reichert Bright-Line hemacytometer (MilliporeSigma), then rested overnight in a 96-well plate at 1 × 10^6^ cells per well.

### In vitro stimulation of allergen-reactive T cells and sorting of AIM^+^ γδ T cells.

Evaluations of allergen-reactive γδ T cell responses were based on a previously described AIM ex vivo assay (24) utilizing CD137 (4-1BB) and CD69 markers. Briefly, after overnight resting PBMCs were stimulated with extracts (10 μg/mL) or HDMAPP (10 μg/mL), or medium alone as negative control, and incubated for 24 hours at 37°C. After the incubation, cells were surface-stained with fluorochrome-conjugated antibodies, for 40 minutes at 4°C, using anti-CD3 (AF700) (UCHT1, Life Technologies, 56-0038-42), anti-CD4 (APCef780) (RPA-T4, Life Technologies, 47-0049-42), anti-CD8 (PerCP-Cy5.5) (RPA-T8, Life Technologies, 45-0088-42), anti-CD14 (V500) (M5E2, BD Biosciences, 561391), anti-CD19 (V500) (HIB19, BD Biosciences, 561121), anti-TCR α/β (PE-DA594) (IP26, BioLegend, 306726), anti-TCRδ1 (FITC) (TS8.2, Life Technologies, TCR2730), anti-TCRδ2 (BV421) (B6, BD Biosciences, 743749), anti-CD137 (APC) (4B4-1, BioLegend, 309810), and anti-CD69 (BV605) (FN50, BD Biosciences, 562989) antibodies. A viability dye (eF506/Aqua) (Invitrogen, 65-0866-18) was also added to discriminate live/dead cells. TotalSeq-C oligonucleotide-conjugated antibodies (BioLegend 394661, 394663, 394665, 394667, 394669, 394671, 394673, 394675, 394677, 394679, 394683, 394685) were also added at this step at 0.01 mg/mL final concentration (1 distinct antibody per sample). After 2 washes in PBS, cells were resuspended into 500 μL of MACS buffer (PBS containing 2 mM EDTA at pH 8.0 and 0.5% BSA) and stored at 4°C until flow cytometry acquisition. AIM^+^ (CD69^+^CD137^+^) Vδ1 and Vδ2 cells were captured by FACS, using a FACSAria Fusion cell sorter (Becton Dickinson), and by employing the gating strategy represented in Supplemental Figure 1. After sorting, ice-cold PBS was added, cells were spun down, and single-cell libraries were prepared as described below. Data collected during sorting were further recorded and analyzed for the frequency of Vδ1 and Vδ2 subsets of AIM^+^ γδ T cells within each stimulated sample using FlowJo X software (version 10) (Tree Star).

### scRNA-Seq data preparation.

For scRNA-Seq, we utilized the 10x Genomics platform. The maximum number of cells sorted from each sample was 20,000 cells–40,000 cells collected in 1.5 mL tubes containing 500 μL of sorting buffer (50% FBS, 50% PBS, 5 μL of recombinant RNase Inhibitor from Takara Bio). For every experiment, we used DNA-oligo–conjugated antibodies as above directed against housekeeping cell surface protein to allow sample multiplexing ranging between 7 and 12 donor samples. In total, we loaded approximately 50,000 sorted cells on the 10x Genomics Chromium Controller per experiment. A total of 9 independent experiments were performed.

Samples were processed using 10x Genomics 5′ end mRNA capture reagents (5v2.0 single cell gene expression profiling chemistry) as per manufacturer’s recommendations; after droplet generation, and in-droplet–based reverse transcription, cDNA was amplified by PCR for 13 cycles; gene expression library preparation followed. After quantification, an equal molar concentration of each library was pooled and sequenced using the NovaSeq 6000 (Illumina) sequencing platform to obtain 28 and 100 bp paired-end reads using the following read length: read 1, 100 cycles; read 2, 100 cycles; i7 index, 10 cycles; and i5 index, 10 cycles. Each gene expression library was sequenced, aiming at a minimum mean sequencing depth of 30,000 reads per cell. Separate libraries were prepared to identify the DNA barcode that would allow demultiplexing and linking each cell to a donor. Briefly, amplified DNA generated from antibody-DNA oligos was separated from transcriptomic cDNA based on size selection following amplification. Antibody-DNA–amplified fragments were less than 300 bp. Library preparation was done in accordance with the manufacturer’s recommendations. Each library was sequenced aiming for 5,000 reads per cell. TotalSeq-C antibodies (BioLegend) were used exclusively to label cells from different donors before pooling them for sequencing. No additional protein profiling alongside gene expression analysis was performed.

### scRNA-Seq analysis — hashtag demultiplexing, quality control, clustering.

Sequencing reads were aligned to the GRCh38 human reference genome using the *multi* pipeline in CellRanger (v5.0) (46). Downstream analyses were performed using the packages Seurat (v5.0.0) (47) and scRepertoire (v1.8.0; see *TCR repertoire analysis*) (48) in R (v4.2.2). Cells with low (≤200) or high (≥5,000) number of features, low (≤1,500) or high (≥20,000) counts, or high mitochondrial content (≥10%) were removed for downstream analysis. Samples were normalized using *SCTransform* (“v2” regularization) (49) with parameters to regress out mitochondrial content and centered log-ratio normalization for the hashtag oligo assay. Hashtag demultiplexing was performed using *MULTIseqDEMUX* (50) with the autoThresh option. Cells identified as “negative” or “doublet” were also removed. Samples were merged and integrated using reciprocal principal component analysis. *FindNeighbors* and *RunUMAP* were performed using 30 dimensions, and *FindClusters* was performed using a resolution of 0.5. Cluster-specific markers were obtained using *FindAllMarkers* with min.pct of 0.25 and logfc.threshold of 0.25. All visualization plots were produced using the packages Seurat, ggplot2, pheatmap, ComplexHeatmap, or GraphPad Prism.

### scRNA-Seq analysis — differential expression, functional enrichment.

DEG analysis between groups was performed using the MAST algorithm (51) on the log-normalized RNA expression matrix using the Seurat *FindMarkers* function with a min.pct of 0.1. A gene was considered significantly differentially expressed if the adjusted *P* value was less than 0.01 and |log_2_ fold change| was greater than 0.3. Functional enrichment analyses were performed using Metascape (27) (http://metascape.org) with GO Biological Processes selected. Module scores were calculated using the Seurat *AddModuleScore* function. The assessment of significant differences between 2 groups in pathway enrichment analysis was performed using Wilcoxon matched pairs signed-rank test between the top 25 pathways per group. The top 25 pathways were chosen after filtering for significantly enriched pathways [log(*q* value) < –1.3] and ranking the resulting list by increasing log(*q* value) and decreasing percentage in GO term.

### TCR repertoire analysis.

TRUST4 (25) was used to reconstruct the γδ TCR repertoire from the scRNA-Seq gene expression data. The provided reference files hg38_bcrtcr and human_IMGT+C were used. Additionally, the parameters --barcodeRange 0 15 + and --read1Range 16 -1 were used. The barcode report was adjusted to 10x format using trust_barcoderep_to_10X for downstream analyses with scRepertoire (v1.8.0).

Only cells containing information for both γ and δ chains were used for downstream analyses. Some genes were listed as having no TRDC (i.e., “None”), in which case “None” was manually replaced with “TRDC” to allow for more accurate representation of clonal expansion, as scRepertoire would count these as distinct genes. Since there are 2 genes for TRGC (i.e., *TRGC1* and *TRGC2*), the “None” values present within the γ chain were not manually replaced, as we could not determine which gene would be correct.

For each of the 5 allergic-stimulation groups, samples with fewer than 5 cells with paired TCR were removed, and the median number of cells was calculated. The nonallergic cohorts had a greater median than their allergic counterparts and therefore were downsampled to the lower median. Samples with more than the median (rounded up if not a whole number) were downsampled to the median, whereas all cells from samples with more than 5 cells but less than the median were used (Supplemental Table 3). Clonal expansion was calculated using “gene” for the parameter cloneCall in *combineExpression* within either each cluster or each cohort. Only the variable genes in the γ and δ chains were utilized to examine the gene pairings (Supplemental Figure 3, H and I).

### Statistics.

Statistical analyses were performed using Kruskal-Wallis test with Dunn’s multiple comparisons test (stimulation-specific cluster proportions), the nonparametric 2-tailed and unpaired Mann-Whitney test (allergic vs. nonallergic proportions), or the Wilcoxon matched pairs signed-rank test (Vδ1 vs. Vδ2 proportions) where appropriate. Prism 10.1.1 (GraphPad) was used for these calculations. Values pertaining to significance are noted in the respective figure, and *P* < 0.05 was defined as statistically significant.

### Study approval.

This study was approved by the Institutional Review Board of La Jolla Institute for Immunology (IRB protocol no. VD-145). Each participant provided written informed consent and was assigned a study identification number with clinical information recorded.

### Data availability.

Supporting data associated with individual values of each figure can be found in the Supporting Data Values Excel file.

The scRNA-Seq data generated and analyzed in this study can be found in NCBI Gene Expression Omnibus (accession number GSE294268). The TCR data generated using TRUST4 (25) are provided as Supplemental Table 6.

This paper does not report original code.

Any additional information required to reanalyze the data reported in this work is available upon request.

## Author contributions

AS and RDSA were responsible for designing research studies. KK, SAL, EDY, AA, EW, SM, RDSA, MM, and GS were responsible for investigation. KK, SAL, RDSA, EDY, and AA were responsible for data analysis. PV and BP were responsible for resources. KK, RDSA, and AS were responsible for manuscript writing. AS, BP, and RDSA supervised. AS was responsible for project administration. AS and RDSA were responsible for funding acquisition.

## Supplementary Material

Supplemental data

Supplemental table 1

Supplemental table 2

Supplemental table 3

Supplemental table 4

Supplemental table 5

Supplemental table 6

Supporting data values

## Figures and Tables

**Table 1:**
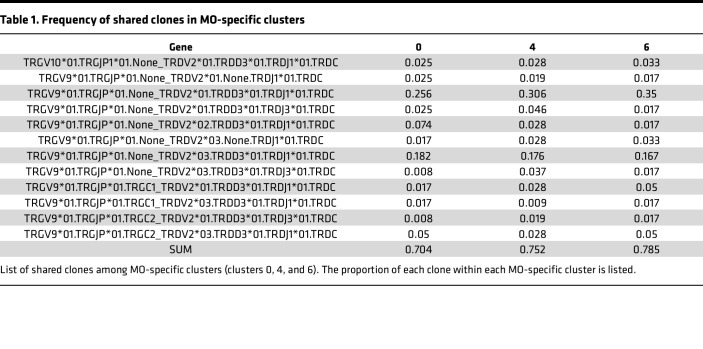
Frequency of shared clones in MO-specific clusters

**Table 2:**
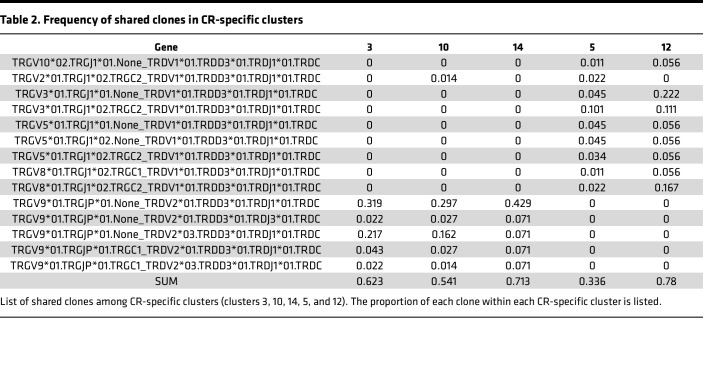
Frequency of shared clones in CR-specific clusters

## Version 1 04/17/2025

In-Press Preview

## Version 2 06/09/2025

Electronic publication
