# Supplementary material for: Single-cell transcriptional analysis reveals allergen-specific signatures in human γδ T cells
Source: JCI Insight. 2025 Apr 17;10(11):e191359. doi: 10.1172/jci.insight.191359 (PMC12220975; doi:10.1172/jci.insight.191359)
Supplement: Supplemental data [file jciinsight-10-191359-s112.pdf]

SUPPLEMENTAL MATERIALS

Supplemental Figures

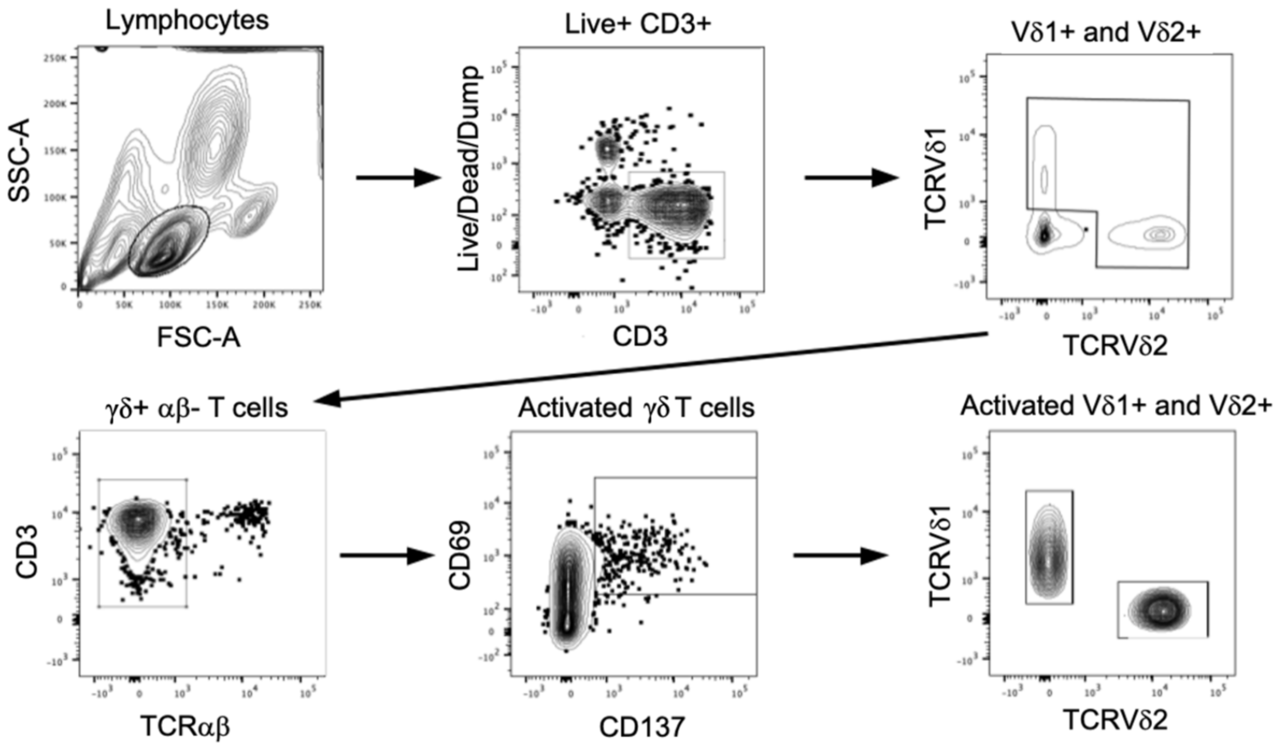

Supplemental Figure 1. Gating strategy, related to Figure 1. Representative gating strategy for sorting AIM+ Vδ1 and Vδ2 cells. Representative dotplots from a CR-stimulated sample is shown.

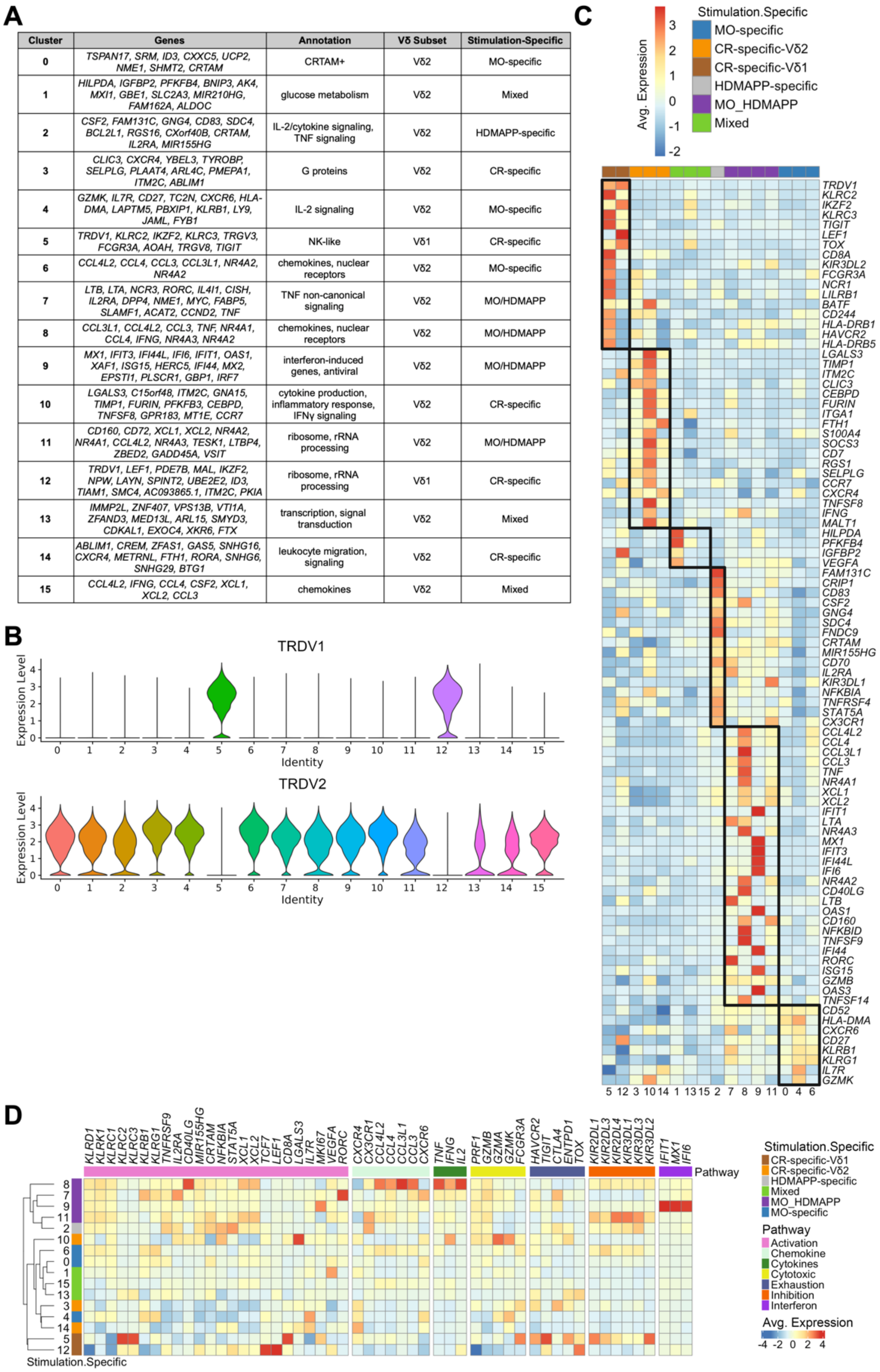

Cluster	Genes	Annotation	Vδ Subset	Stimulation-Specific
**0**	*TSPAN17, SRM, ID3, CXXC5, UCP2, NME1, SHMT2, CRTAM*	CRTAM+	Vδ2	MO-specific
**1**	*HILPDA, IGFBP2, PFKFB4, BNIP3, AK4, MXI1, GBE1, SLC2A3, MIR210HG, FAM162A, ALDOC*	glucose metabolism	Vδ2	Mixed
**2**	*CSF2, FAM131C, GNG4, CD83, SDC4, BCL2L1, RGS16, CXorf40B, CRTAM, IL2RA, MIR155HG*	IL-2/cytokine signaling, TNF signaling	Vδ2	HDMAPP-specific
**3**	*CLIC3, CXCR4, YBEL3, TYROBP, SELPLG, PLAAT4, ARL4C, PMEPA1, ITM2C, ABLIM1*	G proteins	Vδ2	CR-specific
**4**	*GZMK, IL7R, CD27, TC2N, CXCR6, HLA-DMA, LAPTM5, PBXIP1, KLRB1, LY9, JAML, FYB1*	IL-2 signaling	Vδ2	MO-specific
**5**	*TRDV1, KLRC2, IKZF2, KLRC3, TRGV3, FCGR3A, AOAH, TRGV8, TIGIT*	NK-like	Vδ1	CR-specific
**6**	*CCL4L2, CCL4, CCL3, CCL3L1, NR4A2, NR4A2*	chemokines, nuclear receptors	Vδ2	MO-specific
**7**	*LTB, LTA, NCR3, RORC, IL4I1, CISH, IL2RA, DPP4, NME1, MYC, FABP5, SLAMF1, ACAT2, CCND2, TNF*	TNF non-canonical signaling	Vδ2	MO/HDMAPP
**8**	*CCL3L1, CCL4L2, CCL3, TNF, NR4A1, CCL4, IFNG, NR4A3, NR4A2*	chemokines, nuclear receptors	Vδ2	MO/HDMAPP
**9**	*MX1, IFIT3, IFI44L, IFI6, IFIT1, OAS1, XAF1, ISG15, HERC5, IFI44, MX2, EPSTI1, PLSCR1, GBP1, IRF7*	interferon-induced genes, antiviral	Vδ2	MO/HDMAPP
**10**	*LGALS3, C15orf48, ITM2C, GNA15, TIMP1, FURIN, PFKFB3, CEBPD, TNFSF8, GPR183, MT1E, CCR7*	cytokine production, inflammatory response, IFNγ signaling	Vδ2	CR-specific
**11**	*CD160, CD72, XCL1, XCL2, NR4A2, NR4A1, CCL4L2, NR4A3, TESK1, LTBP4, ZBED2, GADD45A, VSIT*	ribosome, rRNA processing	Vδ2	MO/HDMAPP
**12**	*TRDV1, LEF1, PDE7B, MAL, IKZF2, NPW, LAYN, SPINT2, UBE2E2, ID3, TIAM1, SMC4, AC093865.1, ITM2C, PKIA*	ribosome, rRNA processing	Vδ1	CR-specific
**13**	*IMMP2L, ZNF407, VPS13B, VTI1A, ZFAND3, MED13L, ARL15, SMYD3, CDKAL1, EXOC4, XKR6, FTX*	transcription, signal transduction	Vδ2	Mixed
**14**	*ABLIM1, CREM, ZFAS1, GAS5, SNHG16, CXCR4, METRNL, FTH1, RORA, SNHG6, SNHG29, BTG1*	leukocyte migration, signaling	Vδ2	CR-specific
**15**	*CCL4L2, IFNG, CCL4, CSF2, XCL1, XCL2, CCL3*	chemokines	Vδ2	Mixed

Supplemental Figure 2. Cluster profiling, related to Figure 2. A) List of top genes, Vδ subset, and stimulation-specific annotation per cluster. B) Expression levels of TRDV1 (top) and TRDV2 (bottom) per cluster. C) Average expression of top genes per cluster. D) Heatmap of averaged expression of selected genes grouped by associated pathway (x-axis) per cluster (y-axis).

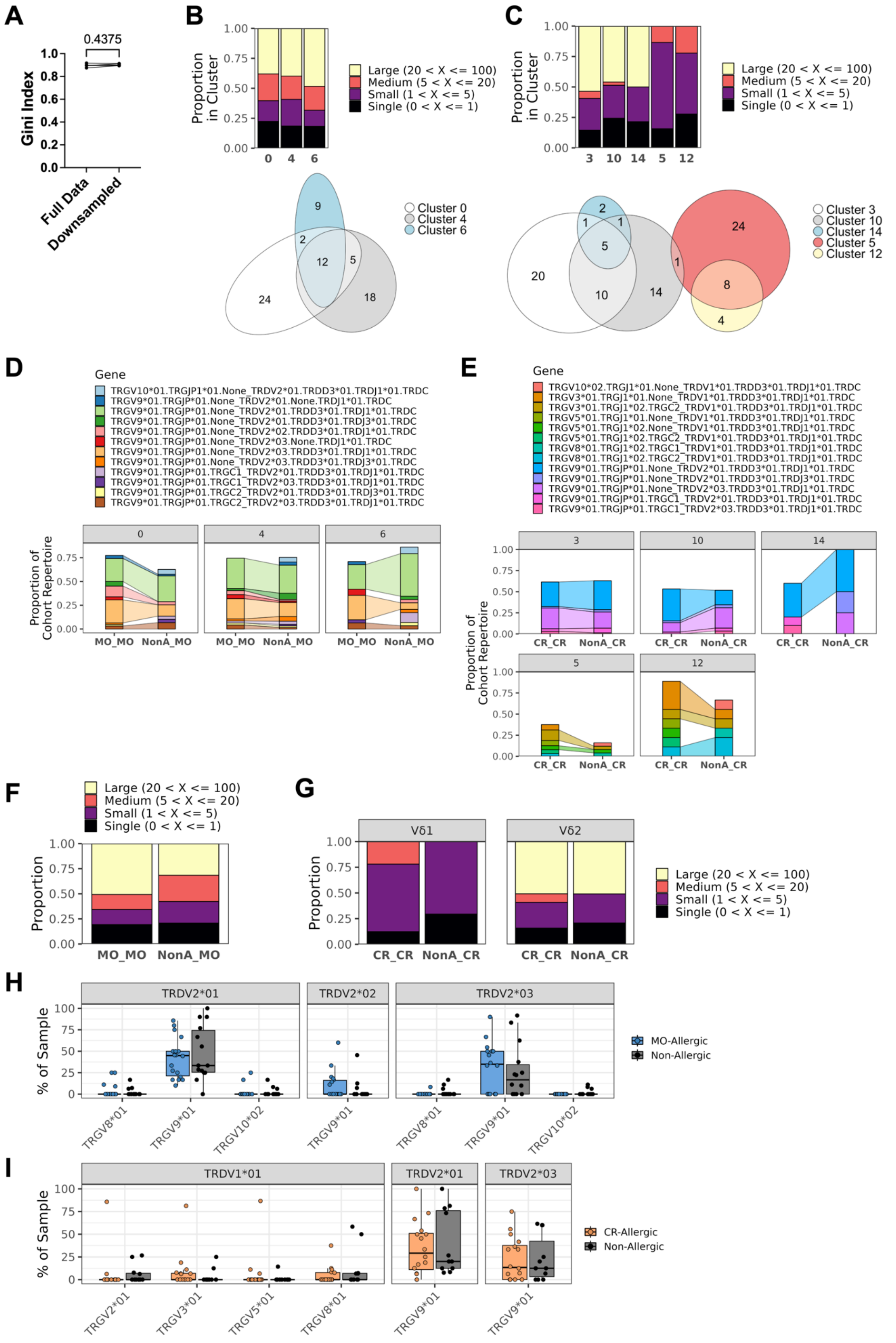
A
B
C
D
E
F
G
H
I
Gini Index
Full Data
Downsampled
0.4375
Proportion in Cluster
Large (20 < X <= 100)
Medium (5 < X <= 20)
Small (1 < X <= 5)
Single (0 < X <= 1)
Cluster 0
Cluster 4
Cluster 6
Cluster 3
Cluster 10
Cluster 14
Cluster 5
Cluster 12
Gene
Proportion of Cohort Repertoire
MO_MO
NonA_MO
CR_CR
NonA_CR
Proportion
Vδ1
Vδ2
% of Sample
TRDV2*01
TRDV2*02
TRDV2*03
TRDV1*01
MO-Allergic
Non-Allergic
CR-Allergic

Supplemental Figure 3. Vγ9Vδ2 dominance and diversity in MO- and CR-specific TCR clusters. A) Gini index for each cohort for the full dataset and the downsampled dataset. Wilcoxon matched-pairs signed rank test. P-values <0.05 considered statistically significant. B,C) Clonal expansion within each MO-specific (B, top) and CR-specific (C, top) cluster. Euler plot showing number of unique clones shared across MO-specific (B, bottom) and CR-specific (C, bottom) clusters. D,E) Proportion of shared clones (Tables 1, 2) within the cohort repertoire of each cluster for MO-specific (D) and CR-specific (E) clusters. Bars connected across cohorts represent the same gene present in both allergic and non-allergic cohorts. F,G) Clonal expansion of allergic vs. non-allergic repertoires in MO-specific (F) and CR-specific (G) clusters. H,I) Percent of each sample with specific TRGV/TRDV pairings separated by allergic status and TRDV gene for MO-specific (H) and CR-specific (I) samples. Only gene pairings found in at least three samples in at least one cohort are shown.

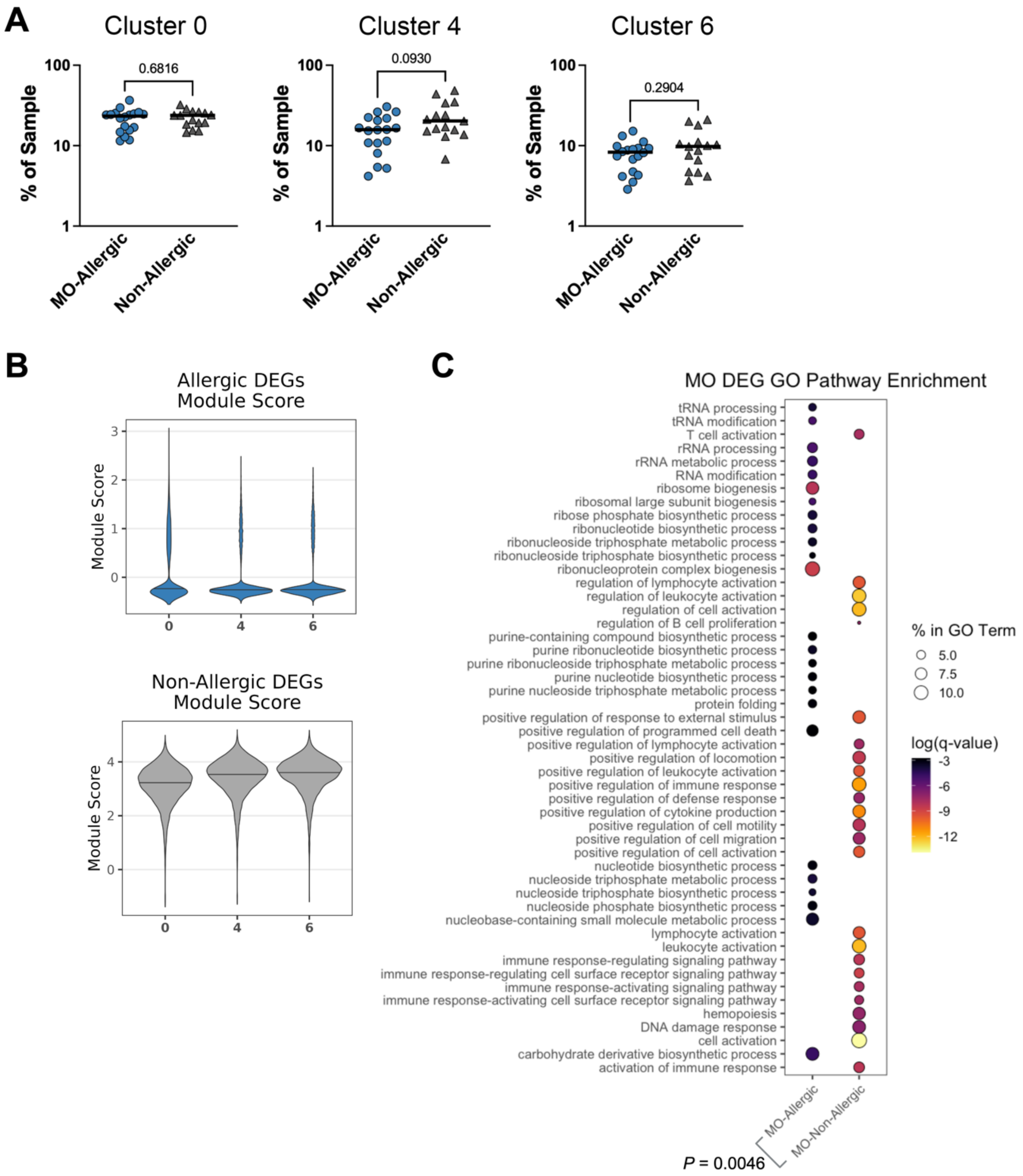

Supplemental Figure 4. MO-specific clusters details, related to Figure 3. A) Frequency of allergic and non-allergic samples within each MO-specific cluster. Mann-Whitney test. B) Module scores per cluster using DEGs associated with allergic (blue) or non-allergic (grey) cohorts depicted in Figure 3. Line at median. C) Top significantly enriched pathways of DEGs found upregulated in MO-specific clusters. The top 25 pathways (log(q-value) < -1.3, then ranked by log(q-value) and % in GO) per group are shown if the threshold of log(q-value) < -1.3 was met. Wilcoxon matched-pairs signed rank test. P-values <0.05 considered statistically significant.

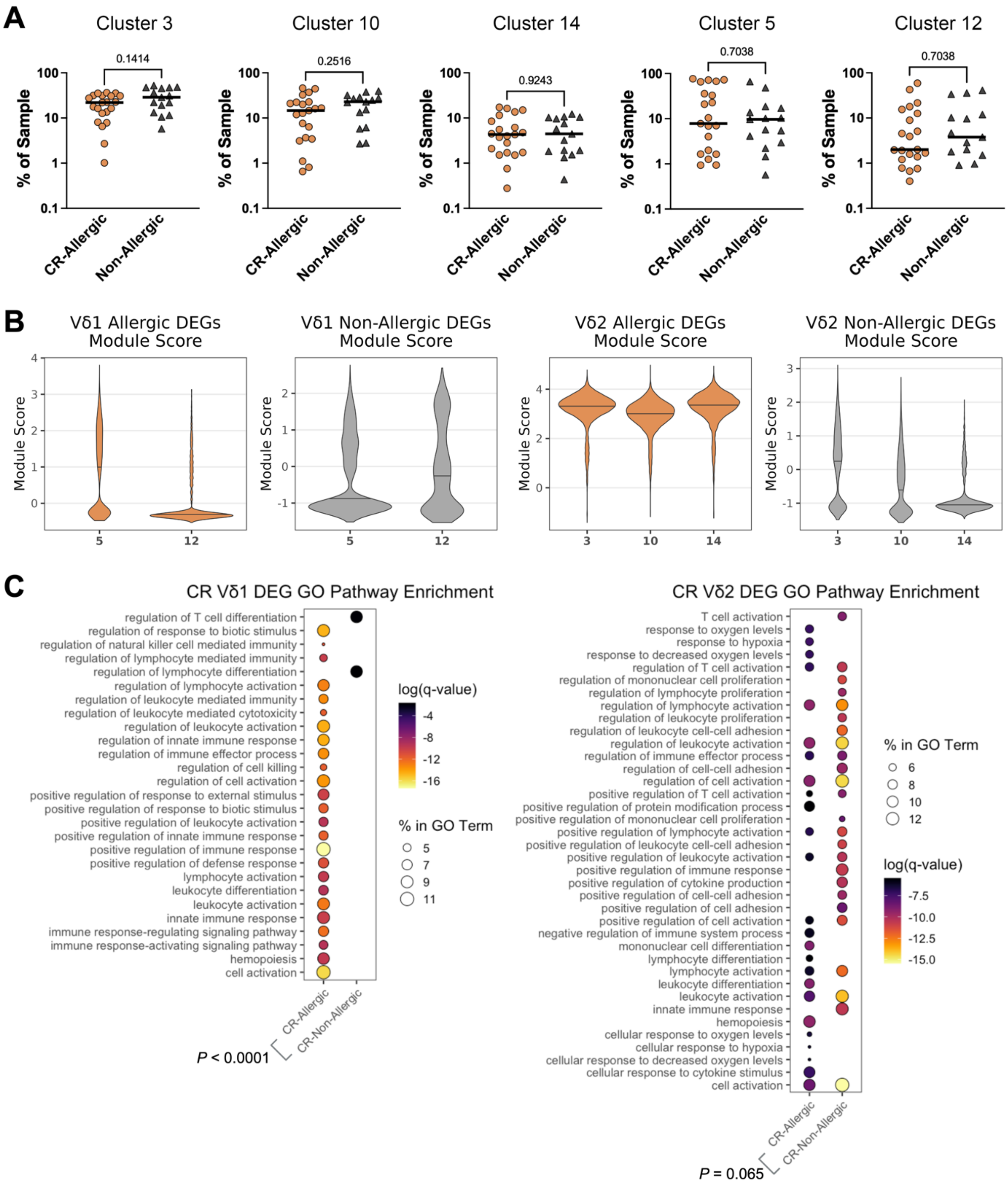

Supplemental Figure 5. CR-specific clusters details, related to Figure 4. A) Frequency of allergic and non-allergic samples within each CR-specific cluster. Mann-Whitney test. B) Module scores per cluster using DEGs associated with allergic (orange) or non-allergic (grey) cohorts depicted in Figure 4. Line at median. C). Top significantly enriched pathways of DEGs found upregulated in Vδ1 (left) or in Vδ2 (right) clusters. The top 25 pathways (log(q-value) < -1.3, then ranked by log(q-value) and % in GO) per group are shown if the threshold of log(q-value) < -1.3 was met. Wilcoxon matched-pairs signed rank test. P-values <0.05 considered statistically significant.

Supplemental Table 1. Donor summary table.

Supplemental Table 2. Cluster markers.

Supplemental Table 3. TCR repertoire information.

Supplemental Table 4. MO-specific clusters DEG in allergic vs. non-allergic cohorts.

Supplemental Table 5. CR-specific clusters DEG in allergic vs. non-allergic cohorts.

Supplemental Table 6. Downsampled TCR repertoire data.
